# The effect of the smartphone app DiaCert on health related quality of life in patients with type 2 diabetes: results from a randomized controlled trial

**DOI:** 10.1186/s13098-022-00965-z

**Published:** 2022-12-17

**Authors:** Madeleine Hummel, Stephanie Erika Bonn, Ylva Trolle Lagerros

**Affiliations:** 1 Clinical Epidemiology Division, Department of Medicine Solna, Karolinska Institutet, Eugeniahemmet T2:02, SE-171 76 Stockholm, Sweden; 2 Obesity Center, Academic Specialist Center, Stockholm Health Services, Stockholm, Sweden

**Keywords:** Diabetes mellitus type 2, Health related quality of life, mHealth, Mobile applications, Randomized controlled trial, Self-management, Smartphone

## Abstract

**Background:**

Type 2 diabetes mellitus is associated with an increased risk of impaired quality of life. Improving health related quality of life (HRQoL) is therefore an important goal in the multimodal management of diabetes. The aim of this study was to evaluate whether the use of the smartphone app DiaCert, that encourage physical activity by promoting daily steps, also impacts HRQoL in patients with type 2 diabetes.

**Methods:**

In this randomized controlled trial, a total of 181 participants with type 2 diabetes were recruited from six health care centers in Stockholm, Sweden. At baseline, participants were randomized 1:1 to the use of the smartphone app DiaCert for a 3 month physical activity intervention in addition to routine care, or to a control group with routine care only. HRQoL was measured using the RAND-36 questionnaire at baseline and at follow-up after 3 months and 6 months. We analysed the HRQoL scores within the intervention and the control groups, respectively, using the Wilcoxon signed-rank test. Between group differences including intervention effect after the 3 month long intervention and after 6 months of follow-up, were assessed using generalized estimating equation models.

**Results:**

In total, 166 participants, 108 men and 58 women, with complete baseline data on RAND-36 were included in analysis. The mean age was 60.2 (SD 11.4) years and the mean Body Mass Index 30.3 (SD 5.4) kg/m^2^. The intervention effect, expressed in terms of the difference in change in HRQoL from baseline to follow-up after 3 months of intervention, showed improvement in the health concept role limitations due to physical health problems (− 16.9; 95% CI − 28.5 to − 5.4), role limitations due to emotional problems (− 13.9; 95% CI − 25.8 to − 2.1), and emotional well-being (− 5.7; 95% CI − 10.4 to − 1.0), in the intervention group compared to the control group. No intervention effect was seen at follow-up after 6 months.

**Conclusions:**

Being randomized to use the smartphone app DiaCert promoting physical activity for 3 months, improved aspects of both physical and emotional HRQoL in patients with type 2 diabetes compared to routine care, but the effect did not last 3 months after the intervention ended.

*Trial Registration* ClinicalTrials.gov Identifier: NCT03053336.

## Background

Type 2 diabetes mellitus is our most common chronic metabolic disease and a growing concern worldwide. This makes it a universal health problem. The global prevalence of diabetes mellitus in the adult population has almost doubled since 1980 [1], and is expected to rise from 8.4% in 2017 to 9.9% in 2045 [2].

The complications of diabetes are numerous and affect both physical and mental health. The majority of patients with diabetes have at least one comorbid chronic disease, where, for example, hypertension, nephropathy, retinopathy, and neuropathy are common [3]. Moreover, type 2 diabetes has been associated with an increased risk of developing depression [4, 5], as well as increased rates of physical disability, including mobility limitations and difficulties with activities of daily living [6]. In a review by Rubin and Peyrot [7], adults with diabetes were found to have an impaired quality of life compared to adults with no chronic disease, partly due to comorbidities and complications of diabetes. Impaired quality of life has in turn been shown to be associated with adverse outcomes, including cardiovascular mortality and total mortality in patients with type 2 diabetes [8]. Therefore, improving health related quality of life (HRQoL), most commonly measured as self-perceived physical, mental, emotional, and social well-being related to health [7, 9], is an important goal in the management of diabetes.

Physical activity has been associated with a decreased risk of complications of diabetes [10, 11]. Moreover, several studies have shown a positive association between physical activity and HRQoL in patients with type 2 diabetes [12–15]. In addition, mHealth, defined by the World Health Organization (WHO) as a medical or public health practice that is supported by mobile devices [16], such as smartphone apps promoting physical activity in adults, have been studied and shown effective in improving different health outcomes [17]. However, previous mHealth studies using app interventions in patients with type 2 diabetes have not targeted physical activity alone, but rather combined management of several aspects such as blood glucose monitoring, weight, diet, and physical activity, or they have included supervised exercise sessions. In addition, only a few of these studies have measured the effect on HRQoL [18, 19], which in patients with type 2 diabetes also has been shown to be associated with regular care and continuity of care [20]. Whether patients’ engagement in their own care using an app with daily physical activity support might also affect the HRQoL is unknown.

To our knowledge, it has not yet been studied if a stand-alone smartphone app intervention with focus on daily physical activity also influence HRQoL in patients with type 2 diabetes. Thus, the aim of this study was to evaluate whether the use of a smartphone app promoting daily steps, impacts HRQoL in patients with type 2 diabetes.

## Methods

### Study design and recruitment of participants

The study design of the DiaCert-study has been described in detail previously [21]. Briefly, the DiaCert-study is a randomized controlled trial of patients with type 2 diabetes. A total of 181 participants were recruited between February 2017 and June 2019 from five primary care centers and one specialized medical center around Stockholm, Sweden. We ended the data collection after almost 2.5 years, since the technical infrastructure of the app could no longer be maintained. Participants were recruited by oral invitation from their treating physician or diabetes nurse during a healthcare visit at one of the participating health care centers. Thereafter, study personnel gave them more information about the study and scheduled them for a baseline meeting. The participants were randomized 1:1 to the use of the smartphone app DiaCert for a 3 month physical activity intervention in addition to routine care, or to a control group with routine care only, i.e., all participants continued their usual care as prescribed by their regular primary care physician and diabetes nurse in accordance with national guidelines. We randomized in blocks of ten by gender and within each health care center, using a random allocation list generated in STATA 14.0. Due to the nature of the intervention, participants were not blinded to their allocation. Inclusion criteria were: a diagnosis of type 2 diabetes, age above 18 years, ability to read and understand Swedish, being able to walk, and having access to and being able to use a smartphone. All participants provided written consent prior to participating in the study. The study was approved by the Regional Ethical Review Board, Stockholm, Sweden.

### Data collection

During the baseline meeting, participant responded to a questionnaire assessing background variables including educational level (≤ 12 years/ > 12 years) and smoking status (never/former/current). At baseline and follow-up after 3 months and 6 months assessment of the primary outcome physical activity (moderate to vigorous physical activity) and secondary outcomes including for example glycated hemoglobin (HbA1c), serum lipids, body weight, blood pressure, and HRQoL were performed. Participants’ weight, height, and waist circumference were measured by study personnel, physical activity was measured using accelerometers and blood samples were collected for measurement of HbA1c and blood lipids. All participants, both the intervention group and the control group, were offered to use the smartphone app DiaCert at the 6 month follow-up.

### The smartphone app DiaCert

The smartphone app “DiaCert” promoted daily steps by registering and displaying the number of steps taken per day, measured by the smartphone. An individual step-goal between 1000 and 10,000 steps per day, based on the participants activity level prior to the study, was set together with the study personnel at baseline. The user could see if the goal was reached each day with the number of steps displayed, as well as a visually displayed circle going from empty (white) to filled (blue). Further, a bar chart with one bar for each day that the app had been used was displayed, where the bar became green if the goal was reached, and red if not. The user also received a positive feedback message in the app when the daily goal was achieved. Every second week, study personnel contacted the participant who was given the opportunity to revise the step goal by an even 500 steps.

HbA1c was measured at baseline, and after the 3 month long intervention and again at the 6 months follow up, i.e., it was not measured during the intervention. All participants, both in the control group and intervention group, received information about their HbA1c levels at baseline and follow-ups from study personal. Participants in the intervention group could also see their baseline HbA1c in the app. The app was compatible with both Android (version 4.1 or higher) and iOS (version 9.2 and higher).

### Measurement of HRQoL

The RAND-36-Item Health survey version 1.0 (distributed by RAND Corporation), referred to as the RAND-36 throughout the rest of the text, is a widely used, self-reporting questionnaire that measures HRQoL [22, 23]. The questionnaire was first published as SF-36 in 1992 [24], and as RAND-36 in 1993 [22]. It is a generic measurement, i.e., it is not population-specific or disease-specific, and it has previously been used in studies including patients with type 2 diabetes [8, 25–27]. The RAND-36 and the SF-36 includes the same items. However, the scoring differs between them regarding the health concepts bodily pain and general health. The difference is often considered negligible as Hays et al. showed that the correlations between the scales were 0.99 in the MOS-study [22].

RAND-36 includes 36 questions (items) about physical and mental health, whereof 35 cover eight health concepts: 1. physical functioning (10 items), 2. role limitations due to physical health problems (4 items), 3. role limitations due to emotional problems (3 items), 4. social functioning (2 items), 5. emotional well-being (5 items), 6. energy/fatigue (4 items), 7. bodily pain (2 items), and 8. general health perceptions (5 items). One additional item measure change in perceived health status today compared to one year ago.

Each item in RAND-36 is scored on a range from 0 to 100 where the score represents the percentage of a total possible score of 100. For example, item 1 has five possible answers, where an answer gives a score of 0, 25, 50, 75, or 100, while item 13 has two possible answers, giving a score of 0 or 100, respectively. Items in the same health concept are averaged together to create the different health concepts scores. Since not all of the health concepts contain the same amount of items, the scores in each health concept are transformed to a 100-point scale ranging from 0 to 100, where higher scores indicate better HRQoL [23, 28].

### Statistical analysis

Baseline characteristics are presented as mean (SD) and n (%) for continuous and categorical variables, respectively. To assess whether there were any statistically significant differences between the intervention group and the control group at baseline, the Mann–Whitney test were performed for continuous and Chi-square test was performed for categorical variables. Nonparametric tests were used, since they do not assume normally distributed data.

**Fig. 1:**
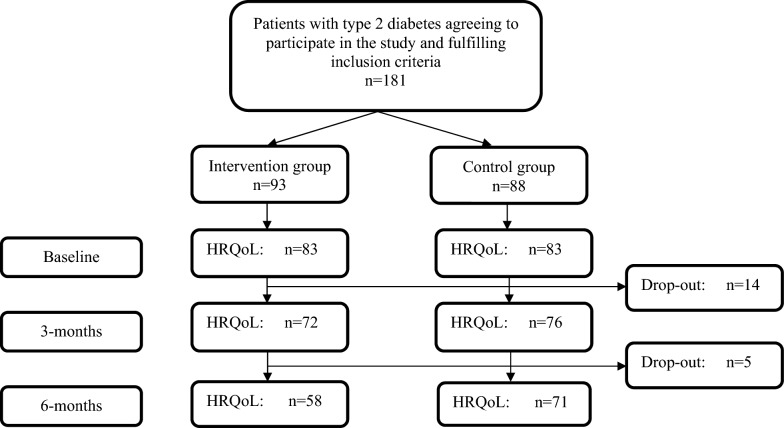
Flow-chart of participation and completeness of RAND-36 data at baseline and follow-up after 3 and 6 months

Only participants with complete baseline data on HRQoL were included in the analysis, fifteen participants were excluded due to missing RAND-36 data at baseline. Within group analysis comparing the HRQoL scores of RAND-36 within the intervention and control groups, respectively, at follow up after 3 months and 6 months were performed using the Wilcoxon signed-rank test. In these analyses, only participants with complete RAND-36 data at baseline and at each follow-up were included. The percentage of missing RAND-36 data at 3 months and 6 months were 11% (18/166) and 22% (37/166), respectively. A flow chart of participants with complete RAND-36 data is presented in Fig. 1.

For in between group analyses, to account for within subject correlations, statistical models for longitudinal data based on generalized estimation equations (GEE) were used to assess both the temporal trend and the intervention effect, and their eventual interaction. The difference in mean between the groups at follow-ups, as well as the intervention effect was analysed. The estimation of intervention effect was expressed in terms of change in HRQoL from baseline between the intervention and control group. We further conducted a sensitivity analysis by only including participants with complete RAND-36 data at baseline and at follow-up at 3 months and 6 months. Adjustments for multiple comparisons were not made [29]. Analyses were performed using Stata 14.2 (Stata Corporation, College Station, TX, USA). The significance level was set to α = 0.05.

## Results

In total, 166 participants were included in the analyses, 108 men and 58 women, with an overall mean age of 60.2 (SD 11.4) years. Characteristics of all participants, and according to intervention group (n = 83) and control group (n = 83), are shown in Table 1. There were no statistically significant differences between the intervention and control group regarding sex, age, body mass index (BMI), waist circumference, smoking status, or educational level. At baseline, a significant lower score for the health concept score energy/fatigue was seen in the intervention group compared to the control group (p = 0.04). There were no statistically significant differences between the groups regarding the other health concept scores at baseline.

**Table 1:** Baseline characteristics of study participants by intervention group and control group

Variable	All (n = 166)	Intervention group (n = 83)	Control group (n = 83)	*p*^a^
	mean (SD)	mean (SD)	mean (SD)	
BMI (kg/m^2^)	30.3 (5.4)	30.1 (5.7)	30.4 (5.2)	0.60
Waist circumference (cm)				
All	107.0 (15.1)	106.6 (15.6)	107.4 (14.7)	0.55
Male	109.7 (14.9)	109.9 (16.3)	109.6 (13.6)	0.74
Female	101.9 (14.3)	101.0 (12.6)	102.9 (16.1)	0.95
HbA1c	53.2 (12.5)	53.7 (13.2)	52.7 (11.8)	0.93
RAND-36 health concepts scores				
Physical functioning	80.2 (20.5)	80.9 (19.6)	79.5 (21.4)	0.71
Social functioning	80.6 (24.0)	77.6 (25.4)	83.5 (22.2)	0.13
Role physical	76.0 (36.9)	74.9 (36.5)	77.1 (37.5)	0.52
Role emotional	76.7 (37.6)	75.0 (37.7)	78.3 (37.7)	0.42
Emotional well-being	72.9 (20.4)	70.2 (22.1)	75.8 (18.2)	0.12
Energy/Fatigue	61.6 (21.3)	58.4 (21.2)	64.9 (21.0)	0.04
Pain	71.8 (27.6)	69.8 (27.3)	73.9 (28.0)	0.24
General health	59.0 (22.6)	58.7 (21.9)	59.2 (23.5)	0.75
Health change	54.8 (22.6)	52.8 (24.0)	56.9 (21.0)	0.40
	n (%)	n (%)	n (%)	
Male	108 (65)	52 (63)	56 (67)	0.52
Age				0.09
< 50 years	32 (19)	19 (23)	13 (16)	
50–59 years	43 (26)	25 (30)	18 (22)	
60–69 years	57 (34)	28 (34)	29 (35)	
> 70 years	34 (21)	11 (13)	23 (28)	
Smoking status				0.29
Never	76 (46)	42 (51)	34 (41)	
Former	68 (41)	29 (35)	39 (47)	
Current	20 (12)	11 (13)	9 (11)	
Missing	2	1 (1)	1 (1)	
Educational level				0.35
≤ 12 years	85 (51)	43 (52)	42 (51)	
> 12 years	79 (48)	38 (46)	41 (49)	
Missing	2	2 (2)	0 (0)	

^a^Mann-Whitney test or Chi-square test

Results from within group analyses are shown in Table 2. HRQoL was significantly higher within the intervention group after 3 months of intervention in three of the health concept scores; emotional well-being (p = 0.02), energy/fatigue (p = 0.02), and health change (p = 0.02). A borderline significant higher score in role limitations due to physical health problems (p = 0.05) was also seen. A significantly lower score for role limitations due to physical health problems was seen in the control group (p = 0.02) after 3 months.

**Table 2:** Within-group difference in HRQoL-scores among intervention and control group at 3 months and 6 months

Intervention group						
RAND-36 health concepts scores	Baseline (n = 72)	3 months (n = 72)	*p*^a^	Baseline (n = 58)	6 months (n = 58)	*p*^a^
	Mean (SD)	Mean (SD)		Mean (SD)	Mean (SD)	
Physical functioning	82.4 (19.4)	83.1 (20.6)	0.68	82.3 (19.7)	80.8 (23.8)	0.93
Social functioning	80.6 (23.8)	86.4 (19.2)	0.08	83.3 (20.4)	85.6 (21.0)	0.10
Role physical	79.2 (32.4)	86.1 (29.4)	0.05	78.1 (33.4)	75.4 (38.5)	0.43
Role emotional	78.6 (35.5)	85.7 (30.9)	0.08	82.1 (33.0)	90.1 (26.7)	0.15
Emotional well-being	73.8 (19.0)	77.5 (17.9)	0.02	76.3 (17.5)	77.9 (19.5)	0.30
Energy/Fatigue	61.3 (19.7)	65.9 (19.2)	0.02	63.3 (19.5)	63.4 (20.4)	0.38
Pain	72.5 (27.0)	72.5 (25.7)	0.80	70.7 (27.7)	73.4 (22.8)	0.30
General health	61.5 (20.7)	63.2 (20.4)	0.14	60.9 (20.3)	62.7 (19.5)	0.23
Health change	53.2 (24.2)	58.7 (21.5)	0.02	55.5 (22.4)	53.9 (22.4)	0.81

Control group						
RAND-36 health concepts scores	Baseline (n = 76)	3 months (n = 76)	*p*^a^	Baseline (n = 71)	6 months (n = 71)	*p*^a^
	Mean (SD)	Mean (SD)		Mean (SD)	Mean (SD)	
Physical functioning	80.0 (21.6)	77.4 (25.3)	0.47	80.0 (20.8)	81.3 (22.3)	0.08
Social functioning	84.0 (22.6)	84.0 (23.1)	0.49	83.9 (22.8)	83.5 (22.7)	0.93
Role physical	76.3 (37.6)	68.2 (41.7)	0.02	75.7 (38.5)	70.2 (39.4)	0.25
Role emotional	77.6 (37.9)	74.2 (40.4)	0.84	77.5 (37.7)	79.0 (36.0)	0.62
Emotional well-being	76.3 (18.1)	74.6 (20.6)	0.60	76.4 (18.3)	76.6 (17.2)	1.00
Energy/Fatigue	65.1 (21.4)	66.3 (21.2)	0.16	64.1 (20.8)	65.0 (19.1)	0.78
Pain	73.5 (28.4)	74.7 (27.4)	0.24	73.0 (28.6)	74.0 (26.2)	0.65
General health	60.4 (23.9)	63.3 (22.9)	0.08	60.2 (23.8)	61.0 (21.2)	0.46
Health change	56.8 (21.2)	57.9 (24.3)	0.53	53.1 (23.7)	59.3 (22.6)	0.67

^a^Wilcoxon signed rank test

Results from between group analysis are shown in Table 3. Participants in the intervention group scored statistically significantly higher than the control group in the health concept role limitations due to physical health problems, with a difference in means of − 14.8 (95% CI − 26.5 to − 3.1) between intervention and control groups at the follow-up after 3 months. This was not seen after 6 months of follow-up. However, the overall trend for role limitations due to physical health problems showed a significant higher score in the intervention group (p = 0.01). No other statistically significant differences were seen.

**Table 3:** The mean differences in HRQoL-scores (control group—intervention group) at 3 months and 6 months

RAND-36 health concepts scores	3 months^a^		6 months^a^		*p* for trend^a^
	Mean difference	(95% CI)	Mean difference	(95% CI)	
Physical functioning	− 4.7	(− 11.6 to 2.3)	− 0.0	(− 7.2 to 7.2)	0.28
Social functioning	− 0.2	(− 7.4 to 7.1)	0.4	(− 7.2 to 7.9)	0.19
Role physical	− 14.8	(− 26.5 to − 3.1)	− 1.8	(− 14.0 to 10.5)	0.01
Role emotional	− 10.8	(− 22.3 to 0.7)	− 7.7	(− 19.8 to 4.3)	0.05
Emotional well-being	− 0.1	(− 6.1 to 5.9)	2.2	(− 4.0 to 8.4)	0.06
Energy/Fatigue	3.1	(− 3.3 to 9.4)	4.9	(− 1.6 to 11.5)	0.40
Pain	3.7	(− 4.6 to 12.1)	2.3	(− 6.3 to 11.0)	0.90
General health	1.6	(− 5.1 to 8.3)	− 0.2	(− 7.1 to 6.6)	0.74
Health change	− 0.52	(− 7.6 to 6.6)	5.4	(− 2.1 to 12.9)	0.28

^a^Calculated using generalized estimating equation

The intervention effect expressed in terms of the difference in change in HRQoL from baseline to follow-up between the intervention and control group at 3 months and 6 months, respectively, is shown in Table 4. The results were significant in three of the health concept scores; role limitations due to physical health problems (− 16.9; 95% CI − 28.5 to − 5.4), role limitations due to emotional problems (− 13.9; 95% CI − 25.8 to − 2.1), and emotional well-being (− 5.7; 95% CI − 10.4 to − 1.0), after 3 months of intervention with improved scores in the intervention group. This was not seen after 6 months of follow-up. However, the overall trend for role limitations due to physical health problems showed a significant improvement in the intervention group (p = 0.01) and a borderline significant improvement for role limitations due to emotional problems (p = 0.05) and emotional well-being (p = 0.06). No other statistically significant difference in change in HRQoL was seen. In complete case sensitivity analyses, including only the 126 participants with complete RAND-36 data at baseline and at follow-up at 3 months and 6 months (data not shown), results remained similar, however, only the intervention effect of role limitations due to physical health problems remained statistically significant (− 14.6; 95% CI − 27.4 to − 1.9) after 3 months.

**Table 4:** Intervention effect, i.e., the difference in change in HRQoL-scores from baseline to follow-up between the intervention and control group, at 3 months and 6 months

RAND-36 health concepts scores	3 months^a^		6 months^a^		*p* for trend^a^
	Intervention effect	(95% CI)	Intervention effect	(95% CI)	
Physical functioning	− 3.1	(− 8.7 to 2.5)	1.6	(− 4.3 to 7.4)	0.28
Social functioning	− 5.9	(− 12.8 to 1.0)	− 5.3	(− 12.6 to 1.9)	0.19
Role physical	− 16.9	(− 28.5 to − 5.4)	− 3.9	(− 16.0 to 8.2)	0.01
Role emotional	− 13.9	(− 25.8 to − 2.1)	− 10.8	(− 23.2 to 1.5)	0.05
Emotional well-being	− 5.7	(− 10.4 to − 1.0)	− 3.4	(− 8.3 to 1.6)	0.06
Energy/Fatigue	− 3.4	(− 8.2 to 1.5)	− 1.5	(− 6.6 to 3.6)	0.40
Pain	− 0.2	(− 7.4 to 7.1)	− 1.6	(− 9.2 to 6.0)	0.90
General health	1.0	(− 3.3 to 5.3)	− 0.8	(− 5.3 to 3.7)	0.74
Health change	− 5.0	(− 12.5 to 2.5)	0.9	(− 6.9 to 8.8)	0.28

^a^Calculated using generalized estimating equation

## Discussion

In this randomized controlled trial in patients with type 2 diabetes, we found an improvement in some aspects of both physical and emotional HRQoL in the group that was randomized to 3 months with the step promoting smartphone app DiaCert, compared to the control group receiving routine care. No long-term effects were seen after 6 months of follow-up, i.e., 3 months after the intervention had ended. Furthermore, being randomized to use the DiaCert-app did not have a statistically significant effect on the primary outcome (moderate to vigorous physical activity) or secondary outcomes of HbA1c, BMI, waist circumference, serum lipids or blood pressure (unpublished data).

The presence of diabetes per se is a known risk factor for impaired HRQoL, but several studies also show that HRQoL is even lower if diabetes complications are present [7, 30]. In a review on HRQoL among patients with diabetes in the Nordic countries [30], the presence of diabetes complications had the greatest impact on HRQoL. Two of the included studies, that comprised both patients with type 1 and type 2 diabetes, found a greater difference in HRQoL between patients with type 2 diabetes and the general population, than of type 1 diabetes and the general population [31, 32]. In another study by Wändell et al. [33], patients with both type 2 diabetes and angina pectoris showed lower HRQoL than those with only angina pectoris. Similarly, HRQoL was found to be lower in patients with diabetes or hypertension, compared to healthy individuals [34].

However, in a meta-analysis by Jing et al. physically active persons with type 2 diabetes had a better HRQoL in five of the included health concepts when measured using the SF-36 questionnaire, compared to less physically active persons. Comparable to our study, the health concept scores role limitations due to physical health problems and emotional well-being were two of the health concepts that showed an effect in the study [35]. This might imply that the physical activity aspect of using the app DiaCert affects these health concepts in a positive way. However, better HRQoL in patients with type 2 diabetes has been shown to be associated with regular care, continuity of care, education by a diabetes nurse, and satisfaction with diabetes education [20]. The daily support from an app might also affect the HRQoL. With a growing diabetes population, strategies to support patients to improve HRQoL are needed.

Several studies have evaluated the effect of apps on health outcomes among patients with diabetes. However, few have studied the effect on HRQoL in patients with type 2 diabetes, and, to the best of our knowledge, even fewer apps have focused on physical activity alone. The majority of apps previously studied are heterogeneous in the functions they provide [18, 19]. In a review by Veazie et al. [18], studies evaluating five commercially available apps for self-management of type 2 diabetes were included, but none of them targeted physical activity. Further, only one of the included studies evaluated the effect on HRQoL, and no difference in change in HRQoL was presented between the intervention and control group [36]. This is in line with another review and meta-analysis by Bonoto et al. [19]. They included six studies on efficacy of apps for patients with type 2 diabetes, where only one study by Holmen et al. [37] measured HRQoL. The patient group in the study by Holmen et al. was similar to ours, consisting of 59%, men with a mean age of 57 years, and a mean BMI of 31.7 kg/m^2^. However, their app did not focus on physical activity alone, but also targeted other aspects of self-management, e.g., diet. They conducted a 3-arm randomized controlled trial (RCT) with two intervention groups, both using the app, while one also received telephone counseling with a diabetes nurse. The control group received routine care. No improvement in HRQoL was seen in any of the three groups after 4 months and 1 year of intervention, respectively [37, 38]. This is in contrast to our study, as we found an effect on some aspect of HRQoL, immediately after 3 months of usage of the app.

Nevertheless, in a recent two group RCT by Coombes et al. [39], 30 participants with type 2 diabetes were randomized to either a 3 month long physical activity intervention, or to a control group receiving usual care. The intervention consisted of weekly exercise sessions and a wrist-worn heart rate monitor connected to a smartphone app, which informed the user if they performed enough physical activity. The sample size in the study by Combes et al. was smaller than ours, but the patient group was similar to ours, consisting of 67% men, with a mean age of 61 years, and a mean BMI of 30.8 kg/m^2^. Comparable to our study, the intervention group had a significant improvement in the health concept score role limitations due to emotional problems, compared to the control group. Long-term effects were however not studied.

The improvements seen in HRQoL at the 3 month follow-up in our study were not maintained after 6 months of follow-up, i.e., 3 months later, when participants had not had access to the app for 3 months. This may suggest that active support from the app is necessary for sustained change. Our results are comparable to the results of a study by van der Weegen et al. [40]. In their 3-armed RCT, patients with chronic obstructive pulmonary disease or type 2 diabetes were randomized to one of three groups. One of the interventions consisted of the use of an accelerometer linked to a smartphone and Web app, combined with physical activity counseling with a nurse, while the other intervention group only received the physical activity counseling. The third group received routine care only. At the end of the intervention, participants receiving the interventions had improved in mental health aspects of HRQoL compared to participants in the group that received routine care only. However, this was not maintained at an additional follow-up 3 months later [40]. Different to our study, the population studied [40] did not only comprise patients with type 2 diabetes, and the use of the app by itself was not studied. Nevertheless, reviews on mHealth interventions in patients with diabetes that have studied the long-term intervention effects have suggested a trend of decreasing intervention effect over time, which is in line with both our results and the results by Weegen et al. [40–42].

A strength of our study is the comparatively large sample size. With 166 participants included in our analyses, our analytical sample is larger or comparable with other studies that have evaluated the effect of apps on HRQoL among patients with type 2 diabetes [36, 37, 39, 40]. Further, participants were recruited from five primary care centers and one specialized medical center located in different areas with diverse populations and levels of socioeconomic status. Our study included a larger number of men compared to women, which reflects the higher prevalence of diabetes type 2 among men, compared to women in Sweden [43–45]. The ratio of men and women, the age of the participants, as well as the recruitment from several primary care centers show external validity, i.e., the generalizability of the study results to the general type 2 diabetes population external to the study population. Moreover, the RAND-36 has previously been used by patients with type 2 diabetes [8, 25–27], and the Swedish version of the RAND-36 has been found to be valid, reliable, responsive, and sensitive [46, 47].

Another strength of our study is the follow up at 6 months, i.e., 3 months after the intervention ended. It is conceivable that 3 months is too short a time for and intervention like this and that daily self-measurement and continuous support using behavioral change techniques is needed to keep the initial improvement in HRQoL over time. However, it should be noted that adherence to the use of the app is unknown, which is a limitation of the study. Furthermore, 22% of the RAND-36 data is missing at follow-up. This is comparable with the follow-up in the study by Holmen et al. where 21% of self-reported data, including HRQoL, was missing [37]. Moreover, in the study by Wayne et al. 35% of the trial completers had missing HRQoL data at the 6 months follow up [36].

Limitations of our study include that the participants were non-blinded to their group allocation. Although the participants in the control group were not aware of what was included in the app during the intervention period, some controls could have found other ways to improve their physical activity, and thereby also affect their HRQoL. Potentially, since participants knew they would receive access to the app at the 6 months follow up, the risk of e.g., using a commercially available app promoting physical activity was reduced.

With a mean age of 60 years, the participants were younger than the general patient with type 2 diabetes in Sweden, with a mean age of 68 years [44, 45]. This may be due to the inclusion criteria of having a smartphone. However, 9 of 10 Swedes use a smartphone regularly [48]. With a growing diabetes population and more people using smartphones, support from an app could be used as a strategy to improve short term HRQoL. However, apps developed for patients should undergo rigorous scientific evaluation.

## Conclusions

In conclusion, being randomized to the use of a smartphone app that encourage physical activity by promoting daily steps for 3 months, in addition to routine care, improved the HRQoL health concepts role limitations due to physical health problems, role limitations due to emotional problems, and emotional well-being in patients with type 2 diabetes, compared to routine care only. However, small differences in effect between the intervention and control group should be interpreted with caution and the effect did not remain at follow-up after 6 months, when the participants had been without the app for 3 months.

## Data Availability

The datasets generated and analysed during the current study are not publicly available due to ethical restrictions, but are available from the corresponding author on reasonable request.

## Abbreviations

BMI: Body mass index
GEE: Generalized estimating equation
HRQoL: Health related quality of life
RCT: Randomized controlled trial
